# 

**Published:** 1829-10-01

**Authors:** 


### BIBLIOGRAPHICAL RECORD.

1. Augusti Caroli Bockii, Medicinæ et Chirurgiæ Doctoris, Prosectoris in Theatro Anatomico Lipsiensi, Accurata Nervorum Spinalium Descriptio. Latine vertit Albertus Fridericus Haenel, Medicinæ et Chirurgiæ Doctor, in Litt, Univ. Lips. Accedunt Tabulæ à Martinio, Med. et Chir. Doct. Delineatæ et à Schroetero in Æs Incisæ. Lipsiæ, MDCCCXXVIII.

---

2. Icones Septem Caroli Augusti Bockii Descriptionum Nervorum Spinalium Illustrantes. Lipsiæ, MDCCCXXVIII.

☞ *These plates of the nerves, which are done upon copper, are extremely accurate, and even minute. The description which accompanies them is lucid and succint, and the work must be acceptable to the anatomist, and indeed to all who wish to keep by them one that will refresh their memory of the nervous system. We return many thanks for the present. We are requested to state that the work may be procured at Messrs. Black, Young, and Young's in this town, or Messrs. Treuttel and Wurtz's.*

---

3. Dispensatorium Homœopathicum. Auctore Dr. Caspario. Denuo Editum, auctum atque Emendatum à Dr. F. Hartmanno. Lipsiæ, sumptibus Baumgaertneri. MDCCCXXIX.

---

4. Bockrivelse over et Menneskelight Misfoster, Hvis Organer Havde et Omvendt Leje. Af J. D. Herholdt, Dr. og Professor, &c. Kjöbenhavn, 1828.

☞ *This is a learned and ingenious work on monstrosities. We return thanks to the talented Professor.*

---

5. Elements of the Theory and Practice of Physic. By George Gregory, M.D. with Notes and Additions, adapted to the Practice of the United States. By Nathaniel Potter, M.D. &c. and S. Colhoun, M.D. Second American from the Third London Edition, with numerous Additions and Amendments. In two Volumes, 8vo, pp. 531—556. Philadelphia, 1829.

---

6. An Experimental Inquiry into the Laws which regulate the Phenomena of Organic and Animal Life. By George Calvert Holland, M.D. &c. 8vo. pp. 470. Edinburgh and London, July 1829.

---

7. An Essay on the Phrenology of the Hindoos and Negroes. By James Montgomery, Esq. Together with Strictures thereon, by Corden Thompson, M.D. Lloyd and Co. Harley Street. 1829.

---

8. Medicine no Mystery; being a brief Outline of the Principles of Medical Science; designed as an Introduction to their General Study as a Branch of a Liberal Education. By John Morrison, M.D. A.B. of Trin. Coll. Dub. 12mo. pp. 165. London, 1829.

☞ *A well-written little work.*

---

9. An Essay on the Deaf and Dumb; shewing the Necessity of Medical Treatment in Early Infancy; with Observations on Congenital Deafness. By John Harrison Curtis, Esq. &c. 8vo. pp. 211. London, 1829.

---

10. Transactions of the Medico-Botanical Society of London. 8vo. stitched, pp. 78. London, 1829. Six Shillings.

---

11. I. Some Observations relating to the Function of Digestion. By A. P. W. Philip, M. D. &c. From the Philosophical Transactions, 1828.

II. Observations on the Functions of the Nervous System. By the same. From the Philosophical Transactions, 1829.

---

12. An Essay upon the Treatment of deep and excavated Ulcer; with Cases. By Rich. Anthony Stafford, M.R.C.S. &c. 8vo. pp. 72. London, 1829. Five Shillings.

---

13. Du Prétendu Eclectisme Médical. Par F. L. V. Broussais, D. M. Discours Préliminaire des Annales de la Médecine Physiologique, pour l'Année, 1829. 8vo. pp. 75; à Paris.

---

14. A Practical Synopsis of Cutaneous Diseases, according to the Arrangement of Dr. Willan; exhibiting a concise View of the Diagnostic Symptoms and the Method of Treatment. By Thomas Bateman, M.D. &c. The Seventh Edition, edited by Anthony Todd Thompson, M.D. F.L.S. &c. 8vo. with a coloured plate, pp. 460. London, 1829.

☞ *An excellent edition of an admirable work.*

---

15. The Art of prolonging Human Life; in which the subject is fully considered both philosophically and practically. By CHRISTOPHER WM. HUFELAND, M. D. First Physician to the King of Prussia, Counsellor of State, &c. A new Edition, with Notes by an English Physician. 12mo. bound; pp. 328. London, 1829.

☞ *A very deserving little volume, and one that will afford both instruction and amusement to the general and professional reader.*

---

16. A Code of Medical Regulations for the Honorable East India Company's Establishment of Surgeons, belonging to the Presidency of Prince of Wales' Island, Singapore, and Malacca. Drawn up at the express desire of Government, by W. E. E. CONWELL, B. L. A.B. & M.D. of Paris. 8vo. stitched, pp. 85. Singapore, 1828.

17. Observations chiefly on Pulmonary Disease in India, and an Essay on the Use of the Stethoscope. By W.E.E. CONWELL, B. L. B. S. &c. Quarto, stitched, pp. 200. Malaccha. Printed at the Mission Press, 1829.

☞ *We return many thanks to Mr. Conwell for these works, which evince great talent, zeal, and practical information. The Essay on the Stethoscope in particular evinces a deep acquaintance with the information to be derived from auscultation, and a philosophical spirit of induction.*

---

18. Pathological and Practical Researches on Diseases of the Brain and the Spinal Cord. By JOHN ABERCROMBIE, M. D. &c. Second Edition, enlarged. 8vo. pp. 476. Edinburgh, 1829.

☞ *We shall notice some of the additions to this excellent work.*

---

19. Regulations to be observed by Candidates previously to their being taken upon Trial for obtaining Diplomas from the College of Surgeons of Edinburgh. Edinburgh, 1829.

---

20. A Practical Treatise on Acute Abdominal and Pelvic Inflammation; containing a comprehensive Clinical View of Inflammation of the Stomach, Bowels, Peritoneum, Uterus, &c. with a certain and expeditious Method of Cure. By DAVID NICHOLAS BATES, Medical Practitioner. 8vo. pp. 136. London, 1829.

---

21. The Mother's Monitor, or Nursery Errors. By JAMES QUILTER RUMBALL, Fellow of the Royal College of Surgeons, &c. 12mo. pp. 121. Wilson, London, 1829.

☞ *A useful little book to those for whom it is intended.*

---

### TO CORRESPONDENTS.

We are glad to perceive by the CARLISLE JOURNAL, for the 29th of August last, that a most respectable meeting has been held in the Town Hall of Carlisle, for the purpose of considering the best means of establishing a public Infirmary for the County. The Right Honourable the Earl of Lonsdale was in the chair, and the Lord Bishop, the High Sheriff, the Mayor, and the Dean of Carlisle, with many other persons of distinction took an active interest in the measure. The following resolutions were agreed to;—1mo. That it is the opinion of this meeting that a Public Infirmary should be established in this County. 2ndo. That the sum of £4489. having been *already* subscribed for the erection of an Infirmary, a Committee be appointed to ascertain what further sums can be obtained for the purpose, and the probable amount of annual subscriptions to be calculated upon. 3tio. That should the funds prove adequate for the proposed undertaking, the Committee be empowered to make the necessary arrangements about site, &c.

We wish the measure every success which it deserves and no doubt will obtain under the auspices of the noble and charitable persons who have taken it in hand. One word to the medical officers;—if the Infirmary is established, remember that it should not be a close borough, but its professional advantages should be made available as widely as possible to the medical circle around.

Many thanks to Mr. Bury. He will find the case in the first Fasciculus of the next Number.

If the Gentleman residing in Devizes (but whose name has escaped the Editor's memory) will commission a friend to call at his house, mentioning the number of the old series which the gentleman was desirous to obtain, a copy of it will be delivered to him, one having been lately procured.

### INDEX.

Abdomen, knife found in the ! .... 199
Abercrombie, Dr. on diseases of the stomach, &c. ....... 1
Aberdeen, regulations, &c. respecting degrees ....... 73
Abscess in the brain after fractured skull ....... 234
Acné, cases of ....... 453
Acupuncturation, cases of ....... 166
Acute hepatitis—"morbid matters" 167
Adam, Dr. on hospital gangrene.... 371
Addison, Dr. on poisons ....... 32
Adherent hernia, criticisms on Mr. Stevens' views of ....... 546
Adipocire in a hydrocele ....... 533
Affection of legs, resembling elephantiasis ....... 272
Agent immédiate du mouvement vital, M. Dutrochet on the...... 49
Ague, masked double tertian, case of 215
Ague, masked, curious case of .... 465
Alexipharmacus' plan for improving the profession ....... 189
Alveolar abscess ....... 414
Amnesia, or loss of language, case of 259
Amputation—abscess in the lungs 162
Amputation, primary, cases of...... 279
Amputation, secondary, cases of .. 281
Amputation for gangrenous ulcer.. 379
Amussat, M. his treatment of varicocele ....... 256
Amussat, M. his mode of stopping hæmorrhage ....... 545
Anatomy bill, Mr. Warburton's .... 218
Anatomy bill, remarks on the withdrawal of the ....... 285
Anatomy and diseases of the teeth, Mr. Bell on ....... 328
Anatomy, ravings of Gracchus on .. 529
Andrew's, St. regulations respecting degrees ....... 74
Aneurism and the new operation, Mr. Wardrop on ....... 46
Aneurism, cure of, by pressure .... 164
Aneurism, false, of the heart; cases and remarks....... 249
Aneurism by anastomosis, ligature for ....... 444
Aneurism, interesting cases of .... 513
Angina pellicularis ....... 169
Angina œdematosa, tracheotomy performed ....... 175
Angus-shire, leaping ague of ...... 151
Annesley, Mr. on hypochondriasis.. 510
Antimony, tartrite of, externally applied ....... 495
Antrum, cancer of the....... 400
Antrum maxillare, disease of the.. 415
Aphonia, intermittent, cases of.... 488
Apoplexy, hypertrophy of the heart in....... 458
Apothecaries' Company, Irish, injustice of the....... 196
Arachnoid membrane, curious condition of the ....... 187
Argenti nitras, Dr. Hood on the.... 211
Armstrong, Dr. on the morbid anatomy of the bowels, &c. ....... 97
Arracan, its medical topography.. 318
Arteriotomy followed by temporal aneurism ....... 523
Artery, ligature of, ultrà tumorem.. 469
Arthrosia atonica, Dr. Wright on.. 173
Ascites, dissection of a case of .... 266
Atrophy from unsuspected causes—singular case of....... 241
Auricle of the heart, ulceration of the 446
Ballingall, Dr. on fractures ...... 226
Ballingall, Dr. on amputations .... 279
Baron, Dr. on iodine ....... 451
Battley, Mr. his liquor cinchonæ .. 484
Bell, Mr. on diseases of the teeth .. 328
Bell, Mr. on diseases of the teeth.. 408
Biett, M. on lichen ....... 567
Biett, M. on herpes ....... 568
Biett, M. on eczema ....... 570
Biett, M. on elephantiasis Arabum 571
Biliary calculi, recent, effects of.... 447
Bill for legalising anatomy, Mr. Warburton's ....... 218
Bleeding, fatal, in puerperal mania 364
Blood, fluid after death, cases of.. 185
Blood, fœtid fluid injected into the . 347
Blood of man, peculiar odorous principle in ....... 483
Blood-letting, fatal effects of, in puerperal affections....... 298
Boivin, Madame, on polypus uteri and pregnancy ....... 571
Bone, bloody tumour of....... 200
Bones of the cranium, disease and removal of the ....... 523
Brachial artery, aneurism of, from bleeding....... 157
Brain, curious and common condition of its arachnoid membrane.. 187
Brain, ramollissement of the ...... 245
Britain, progressive changes of mortality in ....... 423
Brodie, Mr. on aneurism by anastomosis ....... 444
Brodie, Mr. on cysts connected with the liver....... 535
Brown, Dr. on chorea ....... 236

Burke, cerebral development of.... 193
Burne, Dr. on otorrhœa purulenta.. 538
Calcutta, medical society of ...... 314
Calcutta society, transactions of .. 371
Calender, medical; or student's guide ........................ 70
Cancer, origin and nature of...... 394
Cancer of the face................ 395
Cancer, easy fracture of bones in .. 449
Carbuncle, Polish, Dr. Helbich on.. 204
Cardiac disease, simulated by helleborus albus .................. 196
Carditis, acute idiopathic, description of ......................... 248
Carditis simulating chorea ........ 439
Catarrhus vesicæ of old people.... 210
Cazenave, M. on elephantiasis .... 492
Cerebral and spinal irritation, Dr. Darwall on.................. 433
Chinnock, Mr. his case of phlebitis 155
Chorea, fatal case of.............. 236
Chorea, some observations on .... 481
Cinchonæ cordifoliæ, Battley's liquor 484
Cities, medical statistics of........ 426
Climate, Dr. Clark on............. 121
Clinical medicine, remarks on .... 201
Cloquet's anatomy, translated by Dr. Knox....................... 117
Clubs for suicide !................ 430
College of Surgeons of Edinburgh, regulations of the ............... 552
Compound fracture of the skull, case of............................ 234
Compound fracture of the skull—trephining...................... 238
Congenital cataract, operation for.. 474
Constantinople, practice of physic in 142
Consumption, St. John Long's *cures* of.......................... 287
Cooper, Mr. Sam. his edition of the study of medicine............... 91
Corporation monopolies .......... 196
Countries, medical statistics of.... 425
Cramp of the stomach, Dr. Macfarlane on ...................... 489
Cranial bones, disease and removal of the ....................... 523
Croup, epidemic ................. 169
Cynanche trachealis, Dr. Mills on.. 76
Cysts on the convex surface of the liver ....................... 535
Darwall, Dr. on cerebral and spinal irritation .................... 433
Davis, Mr. on the ergot of rye .... 480
Dental gangrene, causes of........ 330
Devon, climate of its south-west coast ....................... 126
Digestion, new theory of.......... 254
Discolouration of the skin, from imperfect heart................ 159
Diseases of the teeth, Mr. Bell on the 408
Diseases of the heart, interesting cases of ...................... 517
Diseases of the heart, instructive cases of ...................... 556
Dislocation of the patella outwards, curious cases of............... 145
Dublin, regulations relating to medical degrees.................. 71
Dupuytren, M. his case of ligature ultrà aneurisma................ 469
Dupuytren, M. on herniæ of the muscles ...................... 566
Dura mater, tumour growing from the ........................ 512
Dutrochet, M. sur l'agent immédiate de la vie ................... 49
Dutrochet, sur l'endosmose et l'exosmose ....................... 49
Dysentery in the East............ 327
Ear, supposed foreign body in the, reflections ..................... 565
Eczema and elephantiasis, M. Biett on ........................... 570
Edinburgh College of Surgeons, regulations of the................ 552
Elephantiasis Græcorum and Arabum ........................ 492
Elliotson, Dr. on the subcarb. of iron in tetanus ..................... 440
Emetic tartar used in 1790, by Dr. Marryat, in large doses ........ 253
Endosmosis and exosmosis, M. Dutrochet on ...................... 49
England, Dr. Clarke on the climate of.......................... 123
England, southern coast of........ 124
England, west of ................ 129
England, superior salubrity of...... 424
Engravings of the nerves from Scarpa, &c. ...................... 108
Epidemic croup ................. 169
Ergot of rye, observations on the .. 480
Erysipelas, with injury of the head 233
Erythema circinnatum, M. Biett on, 504
Excision of nævi ................ 172
External iliac artery tied, with remarks ....................... 513
Extirpation of the tonsil.......... 208
Extirpation, successful, of the uterus ......................... 564
Eye, affections of the ............ 506
Eye-ball, medullary tumour of the.. 397
Eye-lids and orbit, cancer of the .. 397
Face, malignant diseases of the.... 395
Face, medullary tumour of the.... 396
False aneurism of the heart ...... 249
Fels, Dr. on softening of the stomach ....................... 530
Female diseases, Dr. Marshall Hall on ......................... 289
Female diseases, Dr. Gooch on .... 289

Fever, Dr. O'Reardon on.......... 181
Fever at Arracan, characters of the, 320
Fever, &c. Dr. Stoker's observations on........................ 337
Fever, treatment of.............. 349
Fever, pathological appearances in, 353
Fistula in perineo, new mode of treating........................ 265
Fistula of the parotid duct, treatment of.................... 443
Fluidity of the blood after death .. 185
Foramen ovale open, cases of...... 230
Fracture of bones in cancer ...... 449
Fracture, cases of................ 508
Fractures, simple and compound, remarks on ................ 226
France, climate of ............. 130
France, south-east of ........... 132
Gangrene, deceptive appearances of, 228
Gangrene, after operating for popliteal aneurism ............... 263
Gangrene of the teeth, or caries.... 330
Gangrene, hospital, Dr. Adam on .. 371
Gangrenous ulcer, Mr. Leslie on ... 374
Gangrenous ulcer, treatment of ... 377
Gastro-malaxia, Dr. Fels on ...... 530
Genito-urinary organs, neuralgia of the........................ 276
Glasgow, regulations, &c. respecting degrees .................... 73
Gonorrhœal ophthalmia, case of ... 506
Gooch, Dr. on the diseases of women......................... 289
Gooch, Dr. on the disorders of the mind ...................... 359
Gooch, Dr. on the irritable uterus.. 404
Gracchus, his insane letter on anatomy ...................... 529
Gums, diseases of the ............ 412
Guthrie, Mr. on muscæ volitantes, 184
Guthrie, Mr. his lectures at the College of Surgeons .............. 225
Guthrie, Mr. on injuries of the head, 283
Guthrie, Mr. on the ol. terebinth. in iritis ...................... 525
Hæmorrhage, M. Amussat's new mode of stopping ............ 545
Hæmorrhagic state of the intestine, 100
Hæmorrhagic intestine, symptoms of .......................... 105
Hæmorrhagic pleurisy—operation.. 462
Hall, Dr. Marshall, on female diseases....................... 289
Hall, Dr. on puerperal mania ..... 359
Hastings, Dr. his case of ischuria.. 274
Hastings, Dr. on the use of the stethoscope..................... 497
Hastings, Dr. on cysts connected with the liver ............... 536
Hawkins, Dr. on medical statistics, 420
Head-ache, obstinate, removed by erysipelas ................... 473
Health in ancient and modern times 420
Heart, imperfection of the ........ 159
Heart, extensive wound of ........ 165
Heart, false aneurism of the ...... 249
Heart, pus in the cavities of the ... 475
Heart, diseased, interesting cases of, 517
Heart, report on diseases of the ... 556
Heart, great dilatation and hypertrophy of ................... 561
Helbich, Dr. on the Polish carbuncle, 204
Helleborus albus, for simulating cardiac disease ................ 196
Hepatic phthisis, case of ........ 87
Hepatitis, acute, "morbid matters," 167
Hernia, adherent, Mr. Stephens on, 109
Hernia, inflamed, Mr. Stephens on, 113
Hernia, suggestion for a radical cure of .......................... 115
Hernia—testicles absent from the scrotum ..................... 263
Hernia, curious cases of ......... 547
Herniæ of the muscles, M. Dupuytren on ..................... 566
Herpes, M. Biett on.............. 568
Heurteloup, Baron, his instruments 573
Hindoo treatment of diseased spleen, 317
Hood, Dr. on lunar caustic........ 211
Hospital gangrene, Dr. Adam on .. 371
Hospital gangrene, treatment of .. 373
Human anatomy, M. Cloquet's, by Dr. Knox ..................... 117
Humoralists, difficulties for the.... 345
Hydrocele, injection of, followed by peritonitis, &c.................. 174
Hydrocele of spermatic cord, with peritonitis .................... 178
Hydrocele, adipocire in .......... 533
Hypertrophy of left ventricle—ulcers of ileum, curious case of........ 241
Hypertrophy of the heart in apoplexy ...................... 458
Hypertrophy of the heart, extensive, case of ..................... 466
Hypertrophy of the left ventricle, cases of ..................... 557
Hypochondriasis from chronic inflammation of the bowels....... 150
Hypochondriasis, Mr. Annesley on, 510
Ice to the epigastrium, for biliary calculi ...................... 447
Idiopathic carditis, description of.. 248
Ileus, Dr. Abercrombie on ....... 3
Imperforate rectum, treatment of.. 261
Infamy of the Lancet ............ 153
Inflammation of an ovarian cyst ... 258
Inflammatory asthma!............ 517
Influence of climate on chronic diseases, Dr. Clark on ........... 121
Injuries of the head ............. 232
Injuries of the head, cases of...... 238
Injuries of the head, cases of ...... 283
Intermittent, malignant, case of .. 278

Intermittent aphonia, cases of.... 488
Internal strangulation, remarkable case of.................... 247
Intestinal inflammation, terminations of.................... 24
Intestinal irritation, Dr. Hall on .. 291
Intestinal irritation, cases of...... 293
Intestinal irritation, treatment of.. 296
Intestines, chronic diseases of the.. 26
Intestines, sudden protrusion of into scrotum................ 268
Intus-susceptio................ 105
Iodine, Dr. Baron on ............ 451
Irish Apothecaries' Company, injustice of the ................. 196
Iritis, oil of turpentine in ........ 525
Iron, subcarbonate of, in tetanus .. 440
Irritable uterus, Dr. Gooch on the.. 404
Irritable uterus, causes of the...... 405
Irritable uterus, treatment of the.. 406
Irritation, cerebral and spinal, Dr. Darwall on................ 433
Ischuria, remarkable case of...... 274
Italy, climate of ................ 136
John Bull, letter to, on anatomy... 529
Johnson, Dr. James, his case of Imperfection of the heart ........ 159
Johnson, Dr. James, his case of atrophy, &c...................... 241
Kidneys and bladder, extensive disease of.................... 267
Knox, Dr. his translation of Cloquet's anatomy................ 117
Lancet, character of the.......... 153
Lancet, candour of the............ 181
Language, loss of; Dr. Jackson's case...................... 259
Laryngitis—tracheotomy successful, 467
Laudanum, mysterious case of poisoning by .................. 551
Leaping ague of Angus-shire...... 151
Lee, Dr. on phlegmatia dolens .... 380
Leslie, Mr. on gangrenous ulcer.... 374
Lichen, M. Biett on.............. 567
Limosis dyspepsia, Dr. Good on.... 93
Lip, extensive prolongation of its mucous membrane ............ 566
Liquor cinchonæ cordifoliæ, Battley's ...................... 484
Lithontrity—Baron Heurteloup's instruments .................... 573
Lithotomy after fistula ani........ 508
Liver, cysts on its convex surface .. 535
Local malignant disease, Mr. Travers on ...................... 385
Lower lip, cancer of the .......... 398
Lower jaw, cancer of the ........ 399
Lumbar abscess pointing behind the trochanter.................. 270
Lunar caustic, Dr. Hood on ...... 211
Lungs, Dr. Mills on diseases of the, 79
Lupus, cases of.................. 454
Lupus, M. Biett on .............. 504
Lying-in women, disorders of the mind in ........................ 359
Macfarlane, Dr. on cramp of the stomach .......................... 489
Maculæ in children, best treatment of .......................... 173
Madden, Mr. his travels in Turkey, &c.......................... 141
Madden, Mr. on the plague ...... 323
Malignancy, signification of ...... 387
Malignant intermittent, case of.... 278
Malignant diseases, Mr. Travers on, 385
Martin, Jon. his trial ............ 221
Masked tertian.................... 215
Masked ague, curious case of...... 465
Maxillary sinus, disease of the .... 505
Mayo, Mr. his cases of dislocation of the patella...................... 145
Measles and small-pox, Dr. Stoker 357
Measles, bad effect of blood-letting in ............................ 504
Mechanism of parturition, Professor Naegelé on.................... 88
Medical Calendar, or Student's Guide 70
Medical profession, plan for improving the ...................... 189
Medical reporting from societies .. 204
Medical transactions of Calcutta .. 314
Medical statistics, Dr. Hawkins on 420
Medullary cancer, or fungus hæmatodes .......................... 392
Meningea media, wound of the.... 239
Mercury, good effects of, in hospital gangrene ...................... 373
Mind, Dr. Gooch on the disorders of the ............................ 359
Morbid appearances of the trachea, lungs, and heart, Dr. Mills on the, 75
Morbid anatomy of the bowels, &c. Dr. Armstrong's .............. 97
Morbid anatomy, necessary requisites for ...................... 367
Morgan, Mr. on poisons .......... 32
Morphine, experiments on its modus operandi........................ 257
Morphine prescribed in mistake, poisoning from .................. 551
Mouth and fauces, medullary tumour of the ...................... 399
Mouth and fauces, ulcers of the.... 402
Mucous intestinal membrane, diseases of the .................... 22
Mucous membrane of the lip, great prolongation of................ 566
Murder, curious charge of ........ 199
Muscæ volitantes, Mr. Guthrie on.. 184
Naegelé Prof. on the mechanism of parturition .................. 88
Nævi, treatment of .............. 232
Nævus, excision of, from a child 7 weeks old ...................... 172

Nævus maternus, cured by vaccination.............................. 269
Nævus, spontaneous ulceration of a 459
Nares and antrum, cancerous fungus of the.............................. 401
Nerves, engravings of the, from Scarpa, &c. .................... 108
Neuralgia of the genito-urinary organs .............................. 276
Neuralgia, Mr. Bell on............ 417
Neuralgia, some cases of, by Dr. Passagay .......................... 538
Newstead, Mr. on affections simulating inflammations ............ 478
Nice, climate of...................... 134
Nitrate of silver, Mr. Ceely on .... 496
Obstructed and inflamed hernia, Mr. Stephens on .................... 109
Odorous principle in the blood of man .............................. 483
Oil of turpentine in iritis ........ 525
Open foramen ovale, cases of...... 230
Ophthalmia in the East .......... 327
Opium eaters in Turkey. ........ 143
O'Reardon, Dr. on fever .......... 181
Otorrhœa purulenta, Dr. Burne on 538
Otorrhœa purulenta, treatment of.. 541
Ovarian disease, rapid development of 258
Paraplegia, case of .............. 162
Paralysis and head-ache from intestinal worms .................... 563
Paris, lectures, &c. delivered at.... 72
Parotid fistula, M. Roux's mode of treating .......................... 443
Partial palsy cured by strychnine.. 272
Patella dislocated outwards, curious cases of ........................ 145
Pathological researches, Dr. Abercrombie's .......................... 1
Pathological observations, Dr. Stoker's .............................. 337
Pau, climate of.................... 131
Perforation of the stomach, case of 496
Perineal fistula, new mode of treating .............................. 265
Peritonitis, varieties of .......... 14
Peritonitis, erysipelatous.......... 19
Peritonitis, chronic .............. 21
Peritonitis, chronic, Dr. Armstrong on .............................. 106
Pharynx, food in the, for a year and a half .......................... 166
Phlebitis, Mr. Chinnock's case of .. 155
Phlegmatia dolens, Dr. Lee on.... 380
Phrenitis, otorrhœa purulenta mistaken for .......................... 542
Phrenological character of Burke.. 193
Phthisis, recovery from .......... 85
Plague, Mr. Madden's experience in 144
Plague, Mr. Madden on the........ 323
Plague, etiology of the............ 326
Pleurisy, &c. simulated by affections not inflammation .............. 478
Pleuritis hæmorrhagica — operation 462
Poisons, Dr. Addison and Mr. Morgan on ............................ 32
Poisons, mode of action of ........ 35
Poisons, experiments on .......... 42
Poisoning from morphine, prescribed by mistake...................... 551
Poisoning from laudanum, mysterious case .......................... 551
Polish carbuncle, history of the..... 204
Polypi, vesicular and fleshy........ 403
Polypus uteri and pregnancy ...... 571
Popliteal aneurism—operation--gangrene .............................. 263
Porrigo and psoriasis, cases of .... 455
Primary and secondary amputation, cases of .......................... 279
Prostate gland and vesiculæ, diseases of the.......................... 459
Provincial Medical Gazette, remarks on the............................ 152
Puerperal diseases, Dr. Hall on..... 289
Puerperal inflammation, Dr. Hall's treatment of .................... 295
Puerperal peritonitis, Dr. Gooch on 299
Puerperal disease *not* peritonitis .. 303
Puerperal affections, treatment of.. 312
Puerperal mania, Dr. Marshall Hall on ................................ 359
Puerperal mania, danger of ........ 361
Puerperal mania, etiology of ...... 362
Puerperal mania, proximate cause of 364
Puerperal mania, treatment of .... 368
Pulmonary affections, stethoscope in 497
Purpura hæmorrhagica, case of.... 237
Pus in the cavities of the heart .... 475
Pustules of variola punctured by a needle.............................. 148
Ranula treated by the seton ...... 263
Recto-urethral fistula cured by the cautery .......................... 282
Recto-vaginal abscess, mode of laying open.......................... 175
Rectum, imperforate, treatment of.. 261
Regulations of the College of Surgeons of Edinburgh .............. 552
Rheumatic affection of the heart, cases of the ...................... 559
Rigby, Dr. his translation of the "Mechanism of Parturition".... 88
Royal College of Surgeons, lectures at the.............................. 225
Salivary calculus, Mr. Bell on...... 409
Salivation from *bread* pills........ 167
Salt the grand digestive!.......... 254
Scirrhus, structures affected by.... 388
Scirrhus, structure of.............. 389
Scrotum, sloughing of the, curious cases of .......................... 270

Sedatives in puerperal mania....... 369
Sero-enteritis, symptoms of ....... 101
Sero-enteritis, sub-acute........... 102
Seton successful in ununited fracture 468
Sloughy abscess of the thigh and knee-joint, cases of ............ 456
Small-pox, observations on the treatment of ......................... 148
Smith, Mr. on aneurism of the brachial artery .................. 157
Softening of the stomach, memoir on ............................ 530
Spasm of the stomach, Dr. Macfarlane on ........................ 489
Spermatic cord, hydrocele of, with peritonitis ..................... 178
Spermatic artery, ligature of, for varicocele ........................ 256
Spleen, diseases of the ............ 314
Spleen, diseased, treatment of the.. 317
Spontaneous cure of femoral aneurism .......................... 164
St. John Long's cures and casualties 287
Stafford, Mr. on strictures of the urethra ...................... 216
Statistics, medical, Dr. Hawkins on 420
Stephens, Mr. on hernia........... 109
Stephens, Mr. his views of hernia.. 546
Stethoscope, Dr. Hastings on the .. 497
Stoker, Dr. his observations on fever, &c. ....................... 337
Stomach and intestines, Dr. Abercrombie on the ................ 1
Stomach, &c. inflammation of, producing hypochondriasis ........ 150
Strangulated hernia, cases of...... 176
Strangulation, internal, remarkable case of ...................... 247
Strychnine, partial palsy cured by.. 272
Study of Medicine, 3d Edition ... 91
Stuffing for dental gangrene ....... 333
Subclavian tied *beyond* the tumour for aneurism .................. 469
Suicide, proportion of, in different countries ..................... 429
Switzerland, climate of ........... 140
Sycosis menti, M. Biett on......... 502
Syphilitic sore from suckling ...... 465
Tartar-emetic in large doses employed in 1790 ................ 253
Tartar-emetic plasters in chorea .. 483
Tartrite of antimony externally applied ........................ 495
Teeth, necrosis of the ............ 335
Teeth, Mr. Bell on the diseases of the ........................... 408
Temporal aneurism from arteriotomy ......................... 523
Tetanus, subcarbonate of iron in .. 440
Thigh and knee-joint, cases of abscess of the..................... 456
Thompson, Dr. his new theory of digestion ...................... 254
Tic douloureux, remarks on ....... 357
Tongue, cancer of the ............. 399
Tongue, globular tumour of the.... 402
Tonsil, extirpation of the.......... 208
Tooth-ache, applications for ...... 334
Topography, medical, of Arracan.. 318
Trachea, lungs, and heart, Dr. Mills on the........................ 75
Tracheotomy for angina œdematosa 175
Travellers should be abstemious.... 327
Travels in Turkey, by Mr. Madden 323
Travers, Mr. on malignant diseases 385
Trial of Jonathan Martin........... 221
Tubercle, Dr. Armstrong on the formation of ..................... 97
Tumour, bloody, of bone........... 200
Tumour of the dura mater, curious case of ....................... 512
Turkey, Mr. Madden's travels in .. 141
Twining, Mr. on diseases of the spleen 314
Twisting of arteries employed to stop hæmorrhage ................. 545
Typhoid or adynamic fever, Dr. Stoker on ....................... 343
Ulcerated nævus, case of........... 459
Ulceration of the auricle of the heart 446
Ulno-carpal artery, aneurism of, from wound ................. 159
Ununited fracture cured by the seton 468
Urethra, Mr. Stafford on strictures of the ......................... 216
Uterus, irritable, Dr. Gooch on.... 404
Uterus, successful extirpation of the 564
Vaccination, nævus maternus cured by ............................ 269
Varicocele, ligature of the spermatic artery for .................... 256
Vascular engorgement of spleen, symptoms of the ............... 314
Vena saphena major, inflammation of its cellular coat............. 465
Vesicæ, catarrhus, M. Civiale on the 210
Vesiculæ seminales, diseases of the.. 459
Wake, Dr. his evidence on Martin's trial .......................... 223
Warburton, Mr. his bill for anatomy 218
Warburton, Mr. his bill; remarks on its loss ..................... 285
Wardrop, Mr. on aneurism and the new operation ................. 46
Williams, Mr. his evidence on Martin's trial ...................... 222
Worms, intestinal, a cause of paralysis .......................... 563
Wright, Dr. on arthrosia atonica .. 170

